# Life-stage specific predatory efficiency of 2 generalist predators of *Bemisia tabaci* (Hemiptera: Aleyrodidae)

**DOI:** 10.1093/jee/toag151

**Published:** 2026-06-02

**Authors:** Itohan Aigbedion-Atalor, Abdelmutalab G A Azrag, Alvin M Simmons, Jason M Schmidt

**Affiliations:** Department of Entomology, University of Georgia, Tifton, GA, USA; International Centre of Insect Physiology and Ecology (icipe), Nairobi, Kenya; USDA-Agriculture Research Services, U.S. Vegetable Laboratory, Charleston, SC, USA; Department of Entomology, University of Georgia, Tifton, GA, USA

**Keywords:** biological control, functional response, predator–prey interaction, vegetable system, whitefly management

## Abstract

*Bemisia tabaci* (Gennadius) is an economically damaging invasive pest to global vegetable production. Current management strategies rely heavily on insecticides, but increasing levels of resistance raise sustainability concerns. Therefore, strengthening integrated pest management (IPM) programs requires enhancing biocontrol through improved predator–pest interactions. Despite the well-established relevance of functional response modulation by multiple environmental factors, limited research has explored how plant traits and prey life stages interact to influence the efficiency of generalist predators in vegetable systems. To address this gap, we evaluated the functional responses of 2 adult generalist predators, *Hippodamia convergens* (Guérin-Méneville) and *Geocoris punctipes* (Say), against 3 nymphal instars of *B. tabaci* (second, third, and fourth) on 2 vegetable crops: yellow summer squash (*Cucurbita pepo* L.) and green beans (*Phaseolus vulgaris* L.) at 6 prey densities (5, 15, 25, 50, 75, and 100) under laboratory conditions. Results showed that both predators successfully preyed on all nymphal instars. Overall, predation did not differ significantly between host plants but varied among nymphal instars and increased with prey density. Both predators exhibited a Type III (sigmoidal) response to second instars and a Type II (hyperbolic) response to third and fourth instars. Attack rate increased with prey stage, with *H. convergens* showing the highest attack rate on fourth instars. Our findings highlight the importance of prey life stage in determining predator efficiency and support the potential of *H. convergens* and *G. punctipes* as promising biocontrol agents of *B. tabaci*, contributing to the development of more effective IPM strategies for vegetable production systems.

## Introduction

The sweetpotato whitefly, *Bemisia tabaci* (Gennadius) (Hemiptera: Aleyrodidae), remains one of the most pervasive and economically damaging invasive insect pests threatening global agricultural production (Naranjo and Stansly 2010, De Barro et al. 2011, Horowitz et al. 2020, Kavallieratos et al. 2024). *Bemisia tabaci* is currently present on all continents except Antarctica (De Barro et al. 2011, Pan et al. 2021, Naveed et al. 2023), and has become increasingly difficult to manage due to several aspects of its behavior, pest phenology, and the influence of both abiotic and biotic factors (Lin et al. 2007, Farina et al. 2022). Chemical control using synthetic insecticides remains the most widely adopted method for managing populations of this pest (Perring et al. 2018, Sani et al. 2020, Li et al. 2021a, Ding et al. 2024). However, overreliance and continuous use of synthetic chemical insecticides can cause several adverse effects, including the evolution of insecticide resistance (Naveen et al. 2017, Horowitz et al. 2020, Wang et al. 2023). To mitigate the adverse effects of pesticides, integrated pest management (IPM) requires improved synchrony of environmentally sustainable tactics for the long-term control of *B. tabaci* (Stern et al. 1959, Kogan 1998, Barzman et al. 2015).

Within the IPM framework, biological control provides a promising strategy for managing *B. tabaci*. Globally, several natural enemies are reported to effectively suppress *B. tabaci* populations. These include aphelinid parasitoid wasps from the genera *Encarsia* and *Eretmocerus* (Goolsby et al. 1998, 2014, Arnó et al. 2009, Perring et al. 2018, Horowitz et al. 2020), arthropod predators (Gerling et al. 2001, Hagler et al. 2004, Arnó et al. 2009, Kheirodin et al. 2022), entomopathogenic nematodes (Cuthbertson et al. 2003, 2007, Head et al. 2004, Li et al. 2021b) and entomopathogenic fungi (Wraight et al. 2000, Faria and Wraight 2001, Perring et al. 2018). Among these groups of natural enemies, arthropod predators are particularly important due to their feeding behavior, broad ecological adaptability, and potential for immediate pest suppression across diverse agroecosystems. Two such predators, *Hippodamia convergens* Guérin-Méneville (Coleoptera: Coccinellidae) and *Geocoris punctipes* (Say) (Hemiptera: Geocoridae), are widely distributed generalist species commonly found in various cropping systems across the United States. Though these predators frequently co-occur within similar agroecosystems, they represent distinct functional groups characterized by contrasting feeding mechanisms. *Hippodamia convergens* is an actively foraging coccinellid predator that feeds on soft-bodied insects, including aphids and whiteflies (Hagler et al. 2004, Stowe et al. 2021). Both larvae and adults (approximately 2 to 6 mm and 4.3 to 7.3 mm in length, respectively) are highly mobile and possess well-developed chewing mouthparts that enable rapid capture and complete consumption of prey (Hao et al. 2019, Stowe et al. 2021). This mandibulate feeding mode typically supports high attack rates and relatively short handling times, particularly when prey are abundant and easily accessible (Obrycki and Kring 1998, Hagler et al. 2004). In contrast, *Geocoris punctipes* is an active hemipteran stalking predator whose nymphs and adults (approximately 0.8 to 3 mm and 3 to 6 mm in length, respectively) utilize piercing–sucking mouthparts to penetrate prey tissues and extract cellular contents (Tillman and Mullinix 2003, Torres et al. 2004). Compared to coccinellids, this feeding strategy is usually associated with longer handling times per prey item, as the stylets must penetrate prey tissues, and saliva containing digestive enzymes is required to facilitate tissue breakdown before extraction of internal contents (Hagler et al. 2004). These differences in feeding mechanisms can fundamentally influence the extent to which the predators select and exploit different prey life stages, providing a useful framework for evaluating predator–prey interactions and advancing IPM efforts of *B. tabaci.* Although only a limited number of studies have examined the contributions of *H. convergens* and *G. punctipes* to the biological control of *B. tabaci* (Hagler et al. 2004, Kheirodin et al. 2022, 2024, Parkins et al. 2024), a deeper understanding of their predation dynamics is essential for optimizing their use in IPM programs in vegetable production systems.

One mechanistic approach for evaluating predator performance is the functional response, which describes how predator feeding rate changes with prey density (Solomon 1949, Holling 1959, 1966, Murdoch and Oaten 1975). Two key parameters primarily determine variability in predator killing efficiency: the attack rate, which reflects the ability of a predator to search and capture prey, and handling time, which is the total duration taken to feed on prey (Real 1977, Hassell 1978, Milonas et al. 2011). The interaction between these parameters is important and determines the form of the functional response, which is broadly classified into 3 major types: Type I, II, and III (Holling 1959, 1966, Jeschke et al. 2004). A Type I functional response is characterized by a linear increase in consumption rate with increasing prey density (Holling 1959, Jeschke et al. 2004). Conversely, a Type II response exhibits a hyperbolic relationship, where consumption rate increases rapidly at low prey densities but gradually decelerates with increasing prey densities, ultimately reaching a plateau due to constraints imposed by handling time (Jeschke et al. 2002, Dunn and Hovel 2020). A Type III response displays a sigmoidal pattern, in which consumption rate is low at low prey densities, increases rapidly at intermediate densities, and eventually plateaus at high prey densities, often due to prey switching, learning, or behavioral changes in the predator (Holling 1959, Jeschke et al. 2002, Kalinkat et al. 2023). Among these, the Type II response is the most frequently observed in invertebrate predators and is often associated with effective biological control, as agents exhibiting this pattern can maintain high predation rates at low prey densities; however, this response may lead to reduced prey regulation at high densities due to predator saturation. Whereas, Type III responses promote positive density-dependent predation and are associated with greater stability in predator–prey dynamics, thereby enhancing their potential for more effective biological control (Jeschke et al. 2002, 2004, Abrams 2022). Functional response is intrinsically dynamic as several biotic and abiotic factors are known to influence the parameters and overall shape of the response curve. These include prey and predator life stages and sizes (Hassanpour et al. 2011, Uiterwaal and DeLong 2018, Idemudia et al. 2024), availability of alternative prey (Abraços-Duarte et al. 2021, Cicero et al. 2024), temperature (Islam et al. 2021, Kratina et al. 2022, Michalska et al. 2025), relative humidity (Jena et al. 2024), and host plant traits (De Clercq et al. 2000, Rim et al. 2023).

Host plant traits are particularly important in IPM systems because breeding programs often target the integration of plant defense traits to increase resistance to attack from certain herbivores (Peterson et al. 2016, Fortes et al. 2020, Li et al. 2023). Consequently, the expression of these traits can influence the functional response of predatory natural enemies (Peterson et al. 2016). Plant structural traits, such as leaf pubescence and trichome density, can act as physical barriers that impede predator movement, reduce their prey-search and feeding efficiency (De Clercq et al. 2000, Riddick and Simmons 2014, Paspati et al. 2021), while also restricting prey movement and increasing their susceptibility to predation (Xing et al. 2017). In coccinellid predators, moderate to high trichome densities reduce foraging efficiency by physically impeding movement and limiting access to prey (Riddick and Simmons 2014). Similar negative effects also occur in hemipteran predators, including *G. punctipes*, where increased trichome density hinders movement and prolongs the time required to locate and handle prey (Barbour et al. 1993, Riddick and Simmons 2014). In addition to these plant structural effects, chemical defense compounds produced by glandular trichomes, including plant secondary metabolites, can indirectly affect predator foraging behavior by lowering the nutritional quality of prey, thus decreasing predator feeding efficiency (Jalali and Ziaaddini 2017, Teodoro et al. 2020). However, the effects of host plant traits can be context-dependent, varying according to plant species, predator or prey, and specific environmental conditions. Beyond plant-mediated effects, prey life stage and body size are also key determinants of trophic interaction and food-web stability. Generally, predators may show lower attack rates and longer handling times toward very small or very large prey (Aljetlawi et al. 2004, Kratina et al. 2022, Idemudia et al. 2024), and smaller prey are more likely to be protected within leaves bearing high, dense trichomes. Therefore, the effectiveness of predators in suppressing prey populations may depend on the combined effects of prey life stage and host plant traits, which together may affect the functional responses of the predators.

Despite the established relevance of factors influencing the functional response, there is comparatively limited information exploring how host plant traits and prey life stage collectively influence the effectiveness of generalist predators in vegetable cropping systems. Specifically, information is lacking about how these factors influence the predatory efficiency of *H. convergens* and *G. punctipes*, 2 key predators of *B. tabaci*. Understanding these interactions is crucial given the growing emphasis on IPM and the need to optimize predator performance across diverse agroecosystems. Therefore, in this study, we examined the predatory efficiency of adult *H. convergens* and *G. punctipes* when exposed to second, third, and fourth nymphal instars of *B. tabaci* on 2 vegetable crops, yellow summer squash (*Cucurbita pepo* L.) and green beans (*Phaseolus vulgaris* L.). We hypothesized that predation on each prey life stage would increase with prey densities, with *H. convergens* exhibiting higher predation rates than *G. punctipes*. We further predicted that host plant structural traits may influence predator foraging behavior. In addition, we expected predation to be stage-specific, with third and fourth instar nymphs being susceptible and consumed at significantly higher rates than second instar nymphs, as their larger size may increase detectability on the leaves. Finally, we hypothesized that both predators would exhibit a Type II functional response, assuming satiation at higher prey densities due to handling time constraints.

## Materials and Methods

### Host Plants

Two host plants selected for this experiment were the yellow summer squash (*C. pepo*, cultivar Yellow Crookneck) and green beans (*P. vulgaris*, cultivar Caprice Standard Cruiser). These 2 vegetable crops were chosen due to their contrasting morphological characteristics. *Cucurbita pepo* possesses large, densely pubescent leaves with abundant non-glandular and glandular trichomes (Kolb and Müller 2004, Saitta et al. 2022), whereas *P. vulgaris* bears trifoliate leaves that range from glabrous to lightly pubescent, with predominantly non-glandular trichomes that may be straight to hooked (Xing et al. 2017). In addition, both crops differ in their known susceptibility to *B. tabaci* infestations and are widely cultivated in vegetable production systems, particularly in the Southeastern United States (Li et al. 2021a). The seeds of each host plant were grown in plastic pots (10 × 14 × 15 cm) containing Promix Biofungicide Mycorrhizae Growing Medium (Premier Horticulture Ltd, Quebec, Canada). The seedlings were initially maintained in growth chambers at 25 ± 5 °C, 60% RH, and 14:10 h (Light:Dark) photoperiod for 3 wk. They were thereafter transferred into netted insect-rearing cages in a climate-controlled greenhouse [28 ± 2 °C, 60% RH and 14:10 h (Light:Dark) photoperiod] to rear and maintain colonies of *B. tabaci* for the experiments. Seedlings were watered 3 times a week and fertilized once weekly, using Miracle-Gro All-Purpose Plant Food (NPK 20:20:20, Scotts Miracle-Gro Company, Marysville, OH, United States).

### Insect Colonies

The model pest used as prey in this study was *B. tabaci* Middle East-Asia Minor 1 (MEAM 1) cryptic species complex. MEAM 1 is the prevalent cryptic species of *B. tabaci* in the United States of America (Gautam et al. 2020, Li et al. 2021a). The initial population was developed from a pre-established colony reared on cotton plants (*Gossypium hirsutum* L.) within a climate-controlled greenhouse at the University of Georgia Tifton Campus, Tifton, Georgia, United States, for about 7 generations. The collected samples (adult populations) were reared on 3-wk-old *C. pepo* and *P. vulgaris* inside netted insect-rearing cages. The stock culture was intermittently supplemented with field-collected virus-free populations of *B. tabaci* obtained from infested squash (*C. pepo*) plants. Before introduction to the colony, these newly collected individuals were quarantined on healthy, virus-free host plants and monitored for the development of virus-like symptoms. Only cohorts that did not induce visible symptoms were retained and introduced to boost the colony (Marchant et al. 2024). Fresh plants were introduced every 6 to 8 wk to maintain a continuous and healthy population of *B. tabaci* that was representative of the feral population. To ensure proper acclimation, *B. tabaci* colonies were reared on *C. pepo* and *P. vulgaris* for approximately 3 mo before the start of the experiment.

Two generalist predators, *H. convergens* and *G. punctipes*, were selected for this study because they are widely distributed and relatively abundant in cotton and vegetable fields across the United States (Hagler et al. 2004, Kheirodin et al. 2022, 2024, Parkins et al. 2024). In addition, *H. convergens* and *G. punctipes* were chosen to represent 2 distinct functional groups: chewing and piercing–sucking predators, respectively. Adult *H. convergens* were obtained from Nature’s Control (Phoenix, Oregon, United States), while adult *G. punctipes* were collected from untreated (no pesticide applications) agricultural fields at the University of Georgia Tifton Campus experimental research farm, Tift County, United States (31.473306, −83.529037). Both predator species were reared and maintained in aerated transparent plastic containers within a growth chamber under controlled environmental conditions [25 ± 5 °C, 60% RH and 14:10 h (Light:Dark) photoperiod]. Predators were fed *B. tabaci* nymph-infested leaves every 2 d. In addition, a 10% sugar solution absorbed in cotton balls was provided as a supplementary food source. To standardize hunger levels prior to functional response treatment exposure, predators were starved for 48 h, and to prevent dehydration or water stress, they were provided with moistened cotton balls.

### Functional Response Experimental Approach

Under the same controlled environmental conditions delineated in rearing the predators, the functional responses of the 2 adult predatory species, *H. convergens* and *G. punctipes*, were evaluated. Before the start of the experiments, 25 mating pairs of newly emerged adult *B. tabaci* were introduced into BugDorm netted insect-rearing cages (60 × 60 × 60 cm; MegaView Science Co., Ltd, Taichung, Taiwan), each containing 8 potted plants of either *C. pepo* or *P. vulgaris* for 48 h to allow oviposition. After the 48-h ovipositional period, the adults were removed from the cages. The whitefly egg-infested host plants were then maintained under the same rearing conditions in the climate-controlled greenhouse [28 ± 2 °C, 60% RH and 14:10 h (Light:Dark) photoperiod] until the nymphs reached the desired developmental instars (second, third, or fourth). The rationale for including different nymphal instars of *B. tabaci* in this study was to facilitate a detailed assessment of the feeding efficiencies of the predators across the different developmental stages (eg, Idemudia et al. 2024). The eggs and 1st nymphal instars of *B. tabaci* were excluded from the experiments because our preliminary feeding trials revealed minimal or no predation on these life stages by either predator. The second, third, and fourth nymphal instars of *B. tabaci* were identified morphologically based on their developmental biology. The second instar is identified by its small size, whitish-yellow coloration, and flattened body shape (Sani et al. 2020). The third instar is larger and more flattened than the second, with a color ranging from pale to dark yellow (Kedar et al. 2014, Sani et al. 2020). In the fourth instar, the nymphs appear yellowish-white, and the developing red compound eyes are clearly visible through the cuticle (Perring et al. 2018, Abubakar et al. 2022).

The experimental setup consisted of a transparent cylindrical plastic container (8.9 cm diameter × 7.6 cm height) with a fine nylon mesh-covered lid for ventilation. A 6-cm leaf disc of *C. pepo* or *P. vulgaris* (devoid of pests or disease except *B. tabaci*), detached from the whole *B. tabaci* infested plants were placed at the bottom of each container with the abaxial surface facing upward. To prevent desiccation, the leaf disc rested on a thin layer of 2% agar (Islam et al. 2021, Schoeller et al. 2024). Standardized leaf discs were used to minimize plant structural variability by maintaining consistent leaf surface area and arena dimensions across host plants (Jafarian et al. 2022, Cicero et al. 2024, Hung et al. 2025). After placing the leaves under a stereomicroscope (Labomed Luxeo 6Z LED), *B. tabaci* nymphs were carefully counted, using a fine camel-hair brush, and any excess was gently removed to obtain the target densities of the second, third, and fourth instars. Six prey densities, 5, 15, 25, 50, 75, and 100, were established separately for each nymphal instar, on the 2 host plants. The standardized *B. tabaci* densities for each instar on each host plant leaf disc were distributed in different containers, and thereafter, either one adult *H. convergens* or *G. punctipes* was introduced into the experimental arena. Control treatments having the same set-up, but free of predators, were included to check and adjust for natural mortality of *B. tabaci*. The experiment was replicated 10 times for each predator, prey density, nymphal instar, and host plant (for a total of 720 experimental units). The number of *B. tabaci* nymphs consumed was recorded after 24 h, after which each predator was removed from the arenas. Consumed nymphs were not replaced.

### Statistical Analysis

All statistical analyses were completed in R version 4.5.0 (R Core Team 2025). The mortality of *B. tabaci* across all nymphal instars in all 10 control replicates was very low (∼1%); therefore, we did not adjust for control mortality. Overdispersion was detected, so we used a GLM (generalized linear model) with a negative binomial distribution using the glm.nb function from the MASS package to evaluate the overall effects of predators, host plants, and nymphal instars on the number of *B. tabaci* consumed. Initial prey density was included as a covariate to account for any variation in prey availability across replicates. The global model included the main effects of predators, host plants, nymphal instars, and initial prey density, as well as all possible 2-, 3-, and 4-way interactions among these factors. Model selection was performed using the corrected Akaike Information Criterion (AICc) with the dredge function in the MuMIn (Multi-Model Inference) package. The best-supported model included initial prey density, nymphal instars, host plants, predators, and the interaction between initial prey density and nymphal instars. Type III analysis of deviance (likelihood ratio *χ*^2^ tests) was further conducted to assess the significance of each retained predictor. Post hoc comparisons were performed using estimated marginal means (emmeans), with Tukey’s adjustment to determine any differences among treatment combinations.

Functional response analyses were performed using the FRAIR package, following a 3-step workflow (Juliano 2001, Pritchard et al. 2017). First, the type of functional response was determined by fitting a logistic regression to the proportion of prey consumed as a function of the initial prey densities. The sign and significance of the coefficients determine the functional response type. A significant negative first-order (linear coefficient) indicates a Type II functional response. In contrast, a significant positive first-order term (linear coefficient) and a significant negative second-order (quadratic coefficient) term indicate a Type III functional response (Juliano 2001, Pritchard et al. 2017). Once the response type was determined, we fit the functional response models and estimated the parameters using nonlinear least squares regression. Initial parameter starting points were selected based on biologically realistic ranges and informed by visual inspection of the data and exploratory model fitting, following standard approaches for nonlinear functional response models (Pritchard et al. 2017) (Supplementary Table S1). Since prey were not replaced during the 24-h experiments, all models accounted for prey depletion. Type II responses were modeled using the Rogers random predator equation (Rogers 1972):


$$N_{a}=N_{0\;}[1-\exp\left(-a\;(T-\;T_{h}N_{a})\right)]$$

Type III functional responses were modeled using Hassell’s extension of the Rogers random predator equation (Hassell et al. 1977):


$$N_{a}\;=N_{0}\;[1-\exp\left(-\frac{bN_{0}}{1+cN_{0}}(T-T_{h}N_{a})\right)]$$

where *N_a_* is the number of prey consumed, *N*_0_ is the initial prey density offered, *a* is the attack rate of the predator, *T_h_* is the handling time per prey item, and *T* is the total time available to the predator during the experiment (24 h). For the Type III response, *b* is the attack coefficient, and *c* is the density dependence parameter of the attack rate, both of which are constants relating a and *N*_0_ such that *a* = $\frac{bN_{0}}{1+cN_{0}}$.

To ensure consistency and robust estimation of parameter uncertainty, nonparametric bootstrapping (*n* = 1000) was applied to all fitted models using the frair_boot function. The resulting 95% confidence intervals (CIs) were used for parameter interpretation and comparison (Pritchard et al. 2017).

## Results

Overall, both *H. convergens* and *G. punctipes* successfully preyed upon all 3 nymphal instars (second, third, and fourth) of *B. tabaci* on both host plants, *C. pepo* and *P. vulgaris*. Higher-order interactions were not retained in the best-supported model, indicating they did not significantly contribute to variation in predation rates. The host plants also had no significant effect on predation (*χ*^2^ = 0.38; df = 1; *P* = 0.537). In contrast, prey consumption was strongly influenced by initial prey density (*χ*^2^ = 961.65; df = 1; *P* < 0.001), nymphal instars (*χ*^2^ = 135.52; df = 2; *P* < 0.001), and their interaction (*χ*^2^ = 50.66; df = 2; *P* < 0.001). In addition, consumption differed significantly between predators (*χ*^2^ = 6.14; df =1; *P* = 0.013), with *H. convergens* consuming more nymphs (14.2 ± 0.54) than *G. punctipes* (12.4 ± 0.48) across all treatment combinations. Both predators exhibited stage-specific predation patterns, with significantly higher overall consumption recorded on third and fourth instar nymphs compared to second instar nymphs at most prey densities (Supplementary Table S2).

### Functional Response

**Fig. 1.**
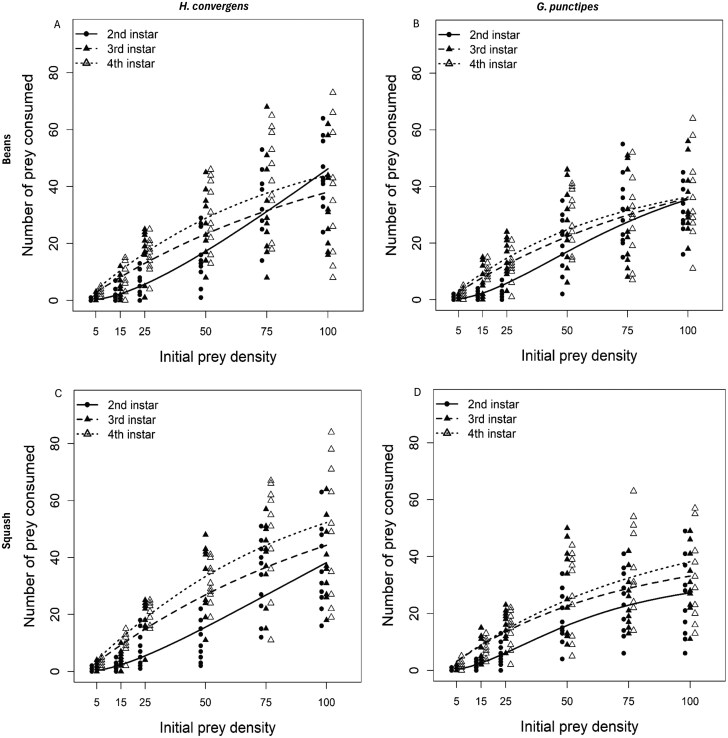
Functional response curves of *H. convergens* and *G. punctipes* preying on 3 nymphal instars (second, third, and fourth) of *B. tabaci* on 2 host plants (Beans and Squash). Each panel shows the number of prey consumed over 24 h as a function of initial prey density. Panels represent predator–host plant combinations: A) *H. convergens* on beans; B) *G. punctipes* on beans; C) *H. convergens* on squash; and D) *G. punctipes* on squash. Within each panel, symbols and lines represent prey instars: second instar (solid line with filled circles), third instar (dashed line with filled triangles), and fourth instar (dotted line with open triangles). Type III responses were observed for second instars, whereas third and fourth instars exhibited Type II responses. Type III responses were fitted using Hassell’s non-replacement model, while Type II responses were fitted using Rogers random predator equation.

Logistic regression analysis showed that both *H. convergens* and *G. punctipes* exhibited a Type III functional response when preying upon second instar *B. tabaci* nymphs across both host plants, as demonstrated by significantly positive linear coefficients followed by significantly negative quadratic coefficients (Table 1). This pattern reflects low consumption at low prey densities, a rapid increase at intermediate densities, and a subsequent deceleration at higher densities, producing sigmoidal response curves (Fig. 1; Supplementary Fig. S1). In contrast, predation on third and fourth instar nymphs followed a Type II functional response, characterized by a significantly negative linear coefficient (Table 1), with consumption increasing steeply at low prey densities and gradually leveling off as prey density increased, resulting in hyperbolic curves (Fig. 1; Supplementary Fig. S1).

**Table 1.** Logistic regression analysis of linear (L) and quadratic (Q) density terms used to determine the type of functional response (Type II or Type III) of *H. convergens* and *G. punctipes* preying on second, third, and fourth nymphal instars of *B. tabaci* across the 2 host plants (Beans and Squash)

Host plants	Predators	Prey instars	Term	Estimates ±1 SE	*Z* value	*P*-value	FR
**Beans**	*G. punctipes*	Second	L	0.083 ± 0.0085	9.72	<0.001***	III
			Q	−0.00058 ± 0.00006	−9.11	<0.001***	
		Third	L	−0.0086 ± 0.0013	−6.35	<0.001***	II
		Fourth	L	−0.013 ± 0.0014	−9.33	<0.001***	II
	*H. convergens*	Second	L	0.048 ± 0.0078	6.24	<0.001***	III
			Q	−0.00026 ± 0.00006	−4.47	<0.001***	
		Third	L	−0.0087 ± 0.0013	−6.49	<0.001***	II
		Fourth	L	−0.014 ± 0.0014	−10.06	<0.001***	II
**Squash**	*G. punctipes*	Second	L	0.061 ± 0.0080	7.58	<0.001***	III
			Q	−0.0005 ± 0.00006	−7.52	<0.001***	
		Third	L	−0.0136 ± 0.0014	−9.92	<0.001***	II
		Fourth	L	−0.012 ± 0.0014	−8.71	<0.001***	II
	*H. convergens*	Second	L	0.037 ± 0.0078	4.81	<0.001***	III
			Q	−0.0002 ± 0.00006	−3.42	<0.001***	
		Third	L	−0.0098 ± 0.0014	−7.26	<0.001***	II
		Fourth	L	−0.014 ± 0.0015	−9.56	<0.001***	II

Following Pritchard et al. (2017), significant positive linear terms (L) combined with significant negative quadratic terms (Q) indicate a Type III functional response, whereas significant negative linear terms alone indicate a Type II functional response. FR indicates the type of functional response determined by the L and Q parameter significance. Estimates (±1 SE) are reported directly from model outputs; variation in decimal places reflects parameter scaling. ***P < 0.001.

Functional response parameters estimated using Hassell’s Type III model revealed no significant differences in attack coefficients (*b*) between predators when feeding on second instar nymphs on the 2 host plants (Table 2). However, the shortest handling time (*T*_h_) was observed for *H. convergens* on beans (0.0008 h), while *G. punctipes* exhibited longer handling times, particularly on squash (0.0282 h). For third and fourth instars, parameters estimated using Rogers Type II model showed that *H. convergens* exhibited the highest attack rate (*a*) when preying on fourth instar nymphs on squash (1.7327 ± 0.1172), compared to *G. punctipes* (Table 3). In contrast, handling time (*T_h_*) did not differ significantly among predator–prey–host plant combinations.

**Table 2.** Functional response estimates for *H. convergens* and *G. punctipes* when preying on second instar *B. tabaci* nymphs on the 2 host plants (Beans and Squash)

Host plants	Predators	*b* (±SE)	95% CI (*b*)	*c* (±SE)	95% CI (*c*)	*T_h_* (±SE)	95% CI (*T_h_*)
**Beans**	*G. punctipes*	0.012 ± 0.002a	0.008–0.017	0.010 ± 0.011a	0 –0.022	0.0178 ± 0.003a	0.011–0.023
	*H. convergens*	0.013 ± 0.005a	0.008–0.020	0.010 ± 0.017a	0–0.025	0.0008 ± 0.011b	0–0.015
**Squash**	*G. punctipes*	0.014 ± 0.003a	0.009–0.021	0.015 ± 0.020a	0–0.014	0.0282 ± 0.011c	0.013–0.039
	*H. convergens*	0.014 ± 0.005a	0.007–0.037	0.016 ± 0.021a	0–0.069	0.0029 ± 0.011d	0–0.021

The parameters were fitted using the Hassell Type III model with non-replacement, *b* = attack coefficient, *c* = density-dependence parameter of the attack rate, and *T_h_* = the handling time during the considered time interval (24 h), and 95% CI = confidence interval. Different lowercase letters within columns indicate significant differences among predators and host plant combinations (*P* < 0.05).

**Table 3.** Functional response estimates for *H. convergens* and *G. punctipes* when preying on third and fourth nymphal instars of *B. tabaci* on the 2 host plants (Beans and Squash)

Host plants	Predators	Prey instars	*a* (±SE)	95% CI (*a*)	*T_h_* (±SE)	95% CI (*T_h_*)
**Beans**	*G. punctipes*	Third	0.8236 ± 0.0650a	0.5263–1.2391	0.0128 ± 0.0019a	0.0014–0.0212
	*H. convergens*	Third	0.8666 ± 0.0652a	0.5694–1.3483	0.0117 ± 0.0017a	0.0010–0.0208
**Squash**	*G. punctipes*	Third	1.0340 ± 0.0815b	0.6977–1.4656	0.0184 ± 0.0016a	0.0109–0.0254
	*H. convergens*	Third	1.0612 ± 0.0725b	0.7226–1.5568	0.0101 ± 0.0013a	0.0023–0.0164
**Beans**	*G. punctipes*	Fourth	1.1747 ± 0.0946c	0.7936–1.6514	0.0170 ± 0.0015a	0.0081–0.0246
	*H. convergens*	Fourth	1.3954 ± 0.0981c	0.9571–2.0529	0.0132 ± 0.0011a	0.0049–0.0214
**Squash**	*G. punctipes*	Fourth	1.0582 ± 0.0794b	0.7701–1.5096	0.0143 ± 0.0015a	0.0064–0.0214
	*H. convergens*	Fourth	1.7327 ± 0.1172c	1.2155–2.3696	0.0110 ± 0.0009a	0.0039–0.0171

The parameters were fitted using the Rogers Type II random predator model with *a* = attack rate, and *T_h_* = the handling time during the considered time interval (24 h), and 95% CI = confidence interval. Different lowercase letters within columns indicate significant differences among predators and host plant combinations (*P* < 0.05).

## Discussion

The functional response of 2 generalist predatory species, *H. convergens* and *G. punctipes*, was evaluated with the rationale of exploiting them for augmentative biocontrol against *B. tabaci*, one of the most challenging pests of vegetable crops. The 2 predators effectively consumed the provided second through fourth nymphal instars of *B. tabaci*. Consistent with our hypothesis, prey consumption increased with initial prey density across the tested prey life stages, with *H. convergens* being highly voracious and consuming more prey on both host plants than *G. punctipes*. This difference may be attributed to variations in the feeding strategies of the 2 predators. *Hippodamia convergens* is a coccinellid beetle that possesses chewing mouthparts and high prey-handling efficiency (Obrycki and Kring 1998), whereas *G. punctipes*, a hemipteran predator, uses piercing–sucking feeding mechanisms that may limit prey intake at high densities (Cohen and Byrne 1992, Hagler et al. 2004). Furthermore, *H. convergens* is known to exhibit strong prey-searching and aggregation responses in dense prey patches (Schellhorn and Andow 2005, Delgado-Ramírez et al. 2019), making it an effective predator under pest outbreak conditions. Additionally, this difference in prey consumption may be partly explained by the predator body size. Adult *H. convergens* are also larger than adult *G. punctipes*, and larger-bodied predators often exhibit a greater visual range, shorter handling times, and higher consumption capacity due to increased energetic demands (Aljetlawi et al. 2004, Vucic-Pestic et al. 2010). This size advantage, together with the more efficient chewing mouthparts of *H. convergens*, may contribute to the higher predation rates observed compared to *G. punctipes*.

Contrary to our initial prediction, prey consumption by both predators was not influenced by host plants used as experimental context. This suggests that these 2 predators may be able to adjust their foraging behavior across different crop habitats, highlighting their potential for use in managing *B. tabaci* in diverse agricultural systems. However, previous studies have shown that host plant traits may impede predator movement, reduce search efficiency, increase handling time, and, in some cases, alter the type of functional response. For example, *Podisus nigrispinus* (Dallas) females exhibited a markedly longer handling time when feeding on fourth instar larvae of *Spodoptera exigua* (Hubner) on tomato (*Solanum lycopersicum* L.) leaves (∼5.6 h) and shorter handling times on eggplant (*Solanum melongena* L.) and sweet pepper (*Capsicum annuum* L.) (∼2.5 to 2.8 h and 3.0 to 3.2 h, respectively) (De Clercq et al. 2000). In that study, a Type II response was observed on sweet pepper and eggplant, but a Type III response on tomato, suggesting that the host plant traits (glandular trichomes and allelochemicals) may interfere with predator performance and reduce foraging efficiency (Riddick and Simmons 2014, Peterson et al. 2016). It is not clear whether the absence of a host plant effect in our study could have resulted from the use of early growth stages of both crop types. Also, leaf trichome density was not quantified, which may have limited our ability to detect differences in plant surface characteristics between host plants, particularly if leaves did not exhibit pronounced variation in trichome density at the stage evaluated. Furthermore, our experiments were conducted in confined arenas, which may have also reduced the host plant-mediated effects on predator foraging behavior. Although leaf-disc systems inherently simplify plant architecture and do not fully capture the structural complexity of whole plants under field conditions, they provide standardized and repeatable environments essential for accurate estimation of functional response parameters prior to field evaluation. By maintaining consistent arena size, leaf area, and prey accessibility across host plants, this approach minimizes confounding variability associated with differences in leaf morphology while preserving plant-specific surface traits that may influence predator–prey interactions. This methodology is widely adopted in most host plant functional response studies as a necessary preliminary step before field validation (Cicero et al. 2024, Schoeller et al. 2024, Hung et al. 2025, Tan and Zemek 2025). Despite the limitation, this work contributes to the relatively few efforts that have directly compared predator performance across different host plants.

For both predators, prey life stage emerged as an important determinant of the magnitude of predation and the functional response types. Both species consumed significantly more third and fourth *B. tabaci* nymphs than second instars, likely reflecting increased detectability and rewarding nutritional benefits of the larger instars, as their biomass and energy content may make them more profitable targets. These findings support our expectation of stage-specific predation; however, contrary to our initial hypothesis of a consistent Type II functional response across all prey life stages, we observed a clear shift in response type depending on prey instar. For *G. punctipes*, a previous study characterized its feeding behavior as conforming to a Holling Type II functional response (Cohen and Byrne 1992). However, our results showed that the functional response of *G. punctipes* is contingent on prey life stage. Specifically, we observed a Type III functional response on the younger nymphs (second instar) and a Type II response on third and fourth *B. tabaci* nymphs. The handling times estimated for *G. punctipes* on both host plants in our study were lower than those reported by Cohen and Byrne (1992), which is likely reflected in the study design differences. Cohen and Byrne (1992) used poinsettia (*Euphorbia pulcherrima* Willd. ex Klotzsch) as the host plant and adult *B. tabaci* as prey, whereas our study focused on nymphal instars.

Similarly, Hagler et al. (2004), reported that *Hippodamia convergens* exhibited a stage-specific feeding pattern on *B. tabaci*, consuming an average of 18 eggs, 6 nymphs, and 10 adults per hour on cotton plants. Although the study did not distinguish between the nymphal instars or estimate the functional response types, it demonstrated clear variation in handling time among prey stages, increasing from approximately 5 s for eggs to 9 s for nymphs and 11 s for adults. Our results corroborate these patterns and extend them by demonstrating that *H. convergens* exhibits a Type III functional response when preying on second instar nymphs and Type II responses on third and fourth instar nymphs, revealing a shared, stage-specific functional response across both predator species. These findings also align with those of Elliott et al. (2011), who investigated the functional response of *H. convergens* on the English grain aphid, *Sitobion avenae* Fabricius (Hemiptera: Aphididae) on wheat (*Triticum aestivum* L.) and reported a Type II response with handling time increasing markedly with aphid developmental stage, from approximately 10.6 s for first instars to 81.5 s for adults. Collectively, these results show that prey life stage plays a significant role in shaping predator foraging behavior and functional response dynamics.

The Type II functional response exhibited by *H. convergens* and *G. punctipes* on the third and fourth nymphal instar of *B. tabaci* is characterized by a decelerating intake rate as prey density increases (Holling 1959, Idemudia et al. 2024). This is typically due to handling time, the time required to pursue, subdue, and consume each prey item, which limits predator consumption capacity (Solomon 1949, Holling 1959, Xiao and Fadamiro 2010). There is substantial information showing that many of the generalist insect predators that show a Type II response on prey, such as the mealybug destroyer, *Cryptolaemus montrouzieri* Mulsant (Coleoptera: Coccinellidae), preying on mealybugs (Rostami et al. 2024), red flower assassin bug, *Rhynocoris segmentarius* (Germar) (Hemiptera: Reduviidae) on feeding on several prey species, including the fall armyworm *Spodoptera frugiperda* (J.E. Smith) (Lepidoptera: Noctuidae) (Idemudia et al. 2024), *Rhynocoris kumarii* Ambrose and Livingston (Hemiptera: Reduviidae) on *Phenacoccus solenopsis* (Tinsley) (Hemiptera: Pseudococcidae) (Sahayaraj et al. 2015), and *Coccinella septempunctata* (L.) preying on aphids (Pervez and Omkar 2005) are considered successful biocontrol agents, due to their high predatory rates. Although Type II responses can be highly effective at suppressing low to moderate prey populations, their efficiency declines at high prey densities due to predator satiation. This limitation can facilitate prey population escape, where rapid prey reproduction outpaces predator consumption (Fernández-Arhex and Corley 2004, Idemudia et al. 2024). Consequently, predators exhibiting Type II responses are most useful in systems where pest populations remain relatively low or where supplemental control measures are applied (Fernández-Arhex and Corley 2004). In addition, such predators are also particularly well-suited for frequent or augmentative release strategies to maintain consistent suppression of pest populations.

Conversely, Type III functional response is typically associated with predators that exhibit prey switching, learning, or increased hunting efficiency at higher prey densities. Type III responses are particularly advantageous in biological control because they confer strong density-dependent regulation. At low densities, reduced attack rates minimize the risk of prey extinction (beneficial for non-target or native prey species), whereas at high prey densities, increased predation pressure effectively suppresses pest outbreaks. This makes Type III predators ideal for maintaining long-term pest suppression and ecosystem stability (Murdoch and Oaten 1975). Although the Type III responses are most often associated with prey switching in multi-prey systems, some studies demonstrate that they can arise in single prey systems when prey is very small, less detectable at low densities, or when the foraging behavior is reduced at low resource availability (Bruzzone et al. 2022, Kratina et al. 2022, Kalinkat et al. 2023). The emergence of a Type III response in our study can be best explained by the prey stage-specific differences. The second instar nymphs are relatively small and likely less detectable on the leaf surface, particularly at low densities, compared to the older nymphs (third and fourth). Their small size may cause them to remain partially concealed within leaf structures, further reducing encounter rates. But as prey density increases, more intensive predator searching may improve detection and access to these early instar nymphs. Additionally, early instar nymphs may also provide lower energetic returns, requiring predators to consume more individuals at low prey densities to meet their nutritional demands.

Considering that both *H. convergens* and *G. punctipes* can occur commonly and at numerically dominant abundances in cotton and diverse vegetable crops (Tillman and Mulrooney 2000, Bowers et al. 2021, Kheirodin et al. 2022, 2024, Parkins et al. 2024, Schmidt et al. 2024), the results of this study emphasize their importance within biological control programs. A previous study using molecular gut content analysis (MGCA) has demonstrated that both predators readily consume *B. tabaci* under field conditions (Kheirodin et al. 2022). Additionally, laboratory and field evaluations by Parkins et al. (2024) indicate that pyriproxyfen can be integrated with *H. convergens* and *G. punctipes*, without imposing significant direct or indirect negative effects on either predator species. Within this context, the current work complements previous studies by providing detailed information on the predatory efficiency of the 2 generalist predators to *B. tabaci* through functional response studies on 2 susceptible vegetable crops. By providing information on the functional response parameters, the study underscores the ability of *H. convergens* and *G. punctipes* to exert stage-specific predation on *B. tabaci* nymphs. Furthermore, integrating field evidence of their abundance and compatibility with certain selective insecticides, these results highlight the potential importance of both predators as promising biological control agents for *B. tabaci* suppression across various vegetable agroecosystems where they co-exist, with clear implications for their integration into IPM programs.

## Supplementary Material

toag151_Supplementary_Data
