# Supplementary material for: Life-stage specific predatory efficiency of 2 generalist predators of Bemisia tabaci (Hemiptera: Aleyrodidae)
Source: J Econ Entomol. 2026 Jun 2;119(4):2599–609. doi: 10.1093/jee/toag151 (PMC13462273; doi:10.1093/jee/toag151)
Supplement: Supplementary file 1 [file toag151_supplementary_data.docx]

**Supplementary Material**

**Life-stage specific predatory efficiency of two generalist predators of *Bemisia tabaci* (Hemiptera: Aleyrodidae)**

**Table S1:** Model parameterization (starting and fixed Values) used to fit Type II (Rogers) and Type III (Hassell) functional response models.

| **Model** | **Parameter** | **Description** | **Value** |
| --- | --- | --- | --- |
| Type II (Rogers) | Attack rate (a) | Starting | 0.5 |
|  | Handling time (T_h_) |  | 0.02 |
|  | Total time to the predator (T) | Fixed | 24 h or 1 day |
| Type III (Hassell) | Attack coefficient (b) | Starting | 0.05 |
|  | Density dependence parameter (c) |  | 0.2 |
|  | Handling time (T_h_) |  | 0.06 |
|  | Total time to the predator (T) | Fixed | 24 h or 1 day |

**Table S2**: Mean (± SE) number of *B. tabaci* nymphs consumed by *G. punctipes* and *H. convergens* across the two host plants, nymphal instars, and prey densities within 24 hours (*n* = 10 per treatment). Different lowercase letters within columns indicate significant differences among predators and nymphal instars combinations at each initial prey density, while different uppercase letters across rows indicate significant differences across initial prey densities at each nymphal instar (*P* < 0.05; Sidak-adjusted pairwise comparisons).

| **Predators** | **Host**  **plants** | **Prey instars** | **Average(±SE) number of prey consumed** | | | | | |
| --- | --- | --- | --- | --- | --- | --- | --- | --- |
|  |  |  | **5** | **15** | **25** | **50** | **75** | **100** |
| *G. punctipes* |  |  | **n =10** | **n =10** | **n =10** | **n =10** | **n =10** | **n =10** |
|  | Beans | 2nd | 0.8 ± 0.20aA | 1.9 ± 0.38aB | 2.5 ± 0.67aB | 19.7 ± 3.27bC | 31.4 ± 3.96abD | 31.8 ± 2.73aD |
|  |  | 3rd | 0.9 ± 0.23aA | 6.6 ± 1.77bB | 14.9 ± 2.14bC | 26.3 ± 4.30cD | 27.4 ± 5.2aD | 34.9 ± 3.88aD |
|  |  | 4th | 2.1 ± 0.48bA | 11.6 ± 0.96cB | 12.4 ± 1.88bB | 28.8 ± 3.22cC | 28.3 ± 4.45aC | 36.7 ± 5.06aD |
|  |  |  |  |  |  |  |  |  |
|  | Squash | 2nd | 0.3 ± 0.15aA | 1.8 ± 0.39aB | 5.4 ± 1.25aC | 18.4 ± 2.92bD | 24.0 ± 3.61aE | 25.3 ± 4.46bE |
|  |  | 3rd | 1.0 ± 0.21aA | 7.3 ± 1.49bcB | 16.0 ± 1.66bcC | 29.2 ± 4.77cD | 24.9 ± 3.05aD | 31.3 ± 3.70aD |
|  |  | 4th | 2.3 ± 0.54bA | 8.3 ± 1.03bcB | 14.1 ± 2.03bC | 27.1 ± 4.53cD | 37.3 ± 5.04bE | 32.6 ± 4.87aDE |
|  |  |  |  |  |  |  |  |  |
| *H. convergens* | Beans | 2nd | 0.7 ± 0.15aA | 2.3 ± 0.62aB | 5.4 ± 1.34aB | 15.2 ± 3.01bC | 35.2 ± 3.75bD | 44.4 ± 3.87cD |
|  |  | 3rd | 1.2 ± 0.33bcA | 4.7 ± 1.19bB | 17.6 ± 2.65bcC | 26.2 ± 3.72cD | 32.4 ± 5.70bD | 34.8 ± 5.20aD |
|  |  | 4th | 3.2 ± 0.51cA | 10.1 ± 1.8cB | 15.7 ± 1.87bcB | 30.4 ± 3.72cC | 43.8 ± 5.20cD | 38.0 ± 7.19aCD |
|  |  |  |  |  |  |  |  |  |
|  | Squash | 2nd | 0.7 ± 0.21aA | 1.7 ± 0.45aA | 7.5 ± 1.93aB | 9.6 ± 2.24aB | 32.9 ± 4.18abC | 36.3 ± 4.62aC |
|  |  | 3rd | 1.8 ± 0.39bA | 4.4 ± 0.91bA | 19.3 ± 2.03cB | 31.3 ± 3.88cC | 39.7 ± 4.15bC | 38.4 ± 4.49aC |
|  |  | 4th | 2.8 ± 0.57cA | 11.1 ± 1.29cB | 21.8 ± 1.23cC | 31.0 ± 2.22cC | 47.5 ± 5.96cD | 50.0 ± 7.49aD |

**Fig. S1**: Functional response curves of *H. convergens* and *G. punctipes* preying on three different nymphal instars of *B. tabaci* on the two host plants (Beans and Squash). Each panel (A and F) shows the number of prey consumed over 24 hours as a function of initial prey density. Solid lines with black circles represent *H. convergens*, and Dashed lines with black open triangles represent *G. punctipes*. Top row: (A) 2nd instar on Beans (Type III); 3rd instar on Beans (Type II); (C) 4th instar on Beans (Type II). Bottom row: (D) 2nd instar on Squash (Type III); (E) 3rd instar on squash (Type II); (F) 4th instar (Type II). Type III responses (A and D) were modeled using Hassell's non-replacement model, while Type II responses (B, C, E, and F) were fitted using Rogers random predator model.
